# Comparing traditional and NGS-based screening strategies for thalassemia in a high-prevalence Hakka population: a population-based study

**DOI:** 10.1038/s41598-026-53002-8

**Published:** 2026-08-18

**Authors:** Haimei Qi, Liqiong Wu, Jie Wang, Jian Xiao, Yulian Zhou, Shilian Chen, Xianping Yuan, Xiaoying Liu, Zhuling Zhang, Haijun Chen, Lin Xiao, JunKun Chen, Wenzhong Li, Shichun Shen, Liyun Song

**Affiliations:** 1 Department of Clinical Laboratory, Ganzhou Women and Children’s Health Care Hospital, Ganzhou, China; 2 Department of Health Examination, Xingguo County Maternal and Child Health Hospital, Ganzhou, China; 3 Department of Clinical Laboratory, Nankang District Maternal and Child Health Hospital, Ganzhou, China; 4 Health Care Department, Xinfeng County Maternal and Child Health Hospital, Ganzhou, China; 5 Department of Clinical Laboratory, Dayu County Maternal and Child Health Hospital, Ganzhou, China; 6 Department of Clinical Laboratory, Xunwu County Maternal and Child Health Hospital, Ganzhou, China; 7 Department of Obstetrics, Ganzhou Women and Children’s Health Care Hospital, Ganzhou, China; 8 Department of Medical Genetics, Ganzhou Women and Children’s Health Care Hospital, Ganzhou, China; 9 Department of Clinical Laboratory, Ganzhou People’s Hospital, Ganzhou, China

**Keywords:** Thalassemia, Next-generation sequencing, Carrier screening, Hakka population, Cost-effectiveness, Diseases, Genetics, Health care, Medical research

## Abstract

Thalassemia is one of the most common monogenic disorders in southern China, with the Hakka population exhibiting a distinct genetic profile. This study evaluated whether next-generation sequencing (NGS)-based universal screening offers advantages over traditional cascade screening in this specific population. We enrolled 91,857 reproductive-age individuals in Ganzhou, a high-prevalence Hakka region, comparing 37,500 participants who underwent traditional screening (2019–2021) with 54,357 who received NGS-based screening. NGS-based screening detected thalassemia carriers at a rate 4.74 times higher than traditional screening (15.05% vs. 3.18%), particularly identifying silent α-thalassemia and β^++^-thalassemia variants that were substantially under-detected by the traditional approach. Crucially, while traditional screening identified 98.7% of β‑high‑severity couples, it detected only 71.4% of α‑high‑severity couples and missed 28.6% of those at risk for Hb Bart’s hydrops fetalis, as well as 32.7% of couples at risk for severe non‑deletional Hb H disease. Traditional screening cost only USD 3.90 per person (versus USD 21.00 for NGS) and provided faster results (6.40 vs. 13.06 days). NGS-based universal screening provides a more accurate assessment of thalassemia burden and genotype diversity, offering critical advantages for prevention programs. Although traditional cascade screening is more cost-effective, its limited sensitivity for α-thalassemia variants leads to substantial underdetection. These findings underscore the need to tailor screening protocols to local epidemiological and genetic characteristics.

**Supplementary Information:**

The online version contains supplementary material available at 10.1038/s41598-026-53002-8.

## Introduction

Thalassemia is a group of hereditary hemolytic anemias caused by mutations or deletions in the globin genes, representing one of the most common monogenic disorders worldwide^1^. The disease exhibits significant geographic clustering, with high-prevalence regions spanning the Mediterranean, the Middle East, the Indian subcontinent, Southeast Asia and South China^2^. Owing to its wide distribution and high carrier frequency, thalassemia has been designated by the World Health Organization as a global priority genetic disease for prevention and control^3^.

As early as the 1950s, Silvestroni and Bianco in Italy highlighted the importance of integrated control programs and advocated for free medical services along with screening and preventive counseling initiatives^4^. Beginning in the 1970s, countries such as Italy, Greece, and Serbia pioneered nationwide prevention programs, thereby setting a precedent for other high-prevalence regions^5^. These efforts have substantially reduced the incidence of severe childhood cases, demonstrating successful global control outcomes^6–8^. Accurate carrier screening and identification are central to building an effective prevention system, with red blood cell indices serving as the initial screening markers^9^. A widely adopted three-step protocol of blood cell analysis, followed by hemoglobin component analysis and then genetic testing, has been promoted since the late twentieth century. It remains the mainstream carrier screening approach in high-prevalence regions worldwide, including southern China^10^.

In recent years, next-generation sequencing (NGS) has been increasingly adopted for genetic screening of reproductive-age populations in high-prevalence areas, such as southern China^11^. Although prior studies have demonstrated that NGS improves carrier detection rates compared with traditional methods^12^, most have focused exclusively on either NGS-based screening or traditional cascade screening, with few direct comparisons performed within the same population. Critically, previous studies have primarily compared carrier detection rates rather than the ultimate goal of screening: identifying couples at high risk of having offspring with clinically severe thalassemia^13^. Furthermore, regional heterogeneity in both carrier frequency and mutation spectrum may lead to considerable variation in the incremental detection yield of NGS over traditional approaches^14^. At present, large-scale population data focusing on specific ethnic groups, including the Han Hakka population, remain limited.

Ganzhou, located in southern Jiangxi Province, China, has a permanent population of 8.97 million, over 95% of whom are of Hakka ethnicity (Supplementary Fig. 1). It is recognized as one of the major ancestral homes of the Hakka people^15^. The carrier profile and mutational spectrum of thalassemia in the Hakka population have been reported to differ from those in other southern Chinese groups^16^. Earlier surveys indicated a thalassemia carrier rate of 9.49% in Ganzhou, while the most recent study reported a rate of 14.55%, the highest in Jiangxi Province^17,18^. Ganzhou was the first area in Jiangxi to implement thalassemia prevention and control programs. Between 2014 and 2021, the traditional three-step cascade screening protocol was carried out among pregnant women in five representative counties of Ganzhou (Xunwu, Dayu, Nankang, Xinfeng, Xingguo). Subsequently, from 2019 to 2022, an NGS-based universal screening program was implemented among premarital examination populations across all 18 counties of Ganzhou.

This study analyzed data from the five counties where both screening strategies were implemented between 2019 and 2021. Unlike previous studies that simply compared carrier detection rates, we performed a comprehensive comparison of the two strategies across multiple clinically relevant dimensions: detailed genotype distribution, identification of at-risk couples stratified by clinical severity, screening costs, and turnaround times. Through this direct comparison in a high-prevalence, ethnically distinct setting, we aimed to identify the underlying causes of detection discrepancies and provide evidence-based insights for optimizing thalassemia prevention strategies.

## Materials and methods

### Study population

This study enrolled 91,857 participants who underwent thalassemia screening between 2019 and 2021 across five counties of Ganzhou Municipality (Nankang, Dayu, Xinfeng, Xingguo, and Xunwu). The screening program included both prenatal and premarital pathways. For pregnant women and their spouses, the traditional three-step cascade screening protocol was applied, involving complete blood count (CBC), hemoglobin electrophoresis (HE), and thalassemia gene detection via PCR-based flow hybridization. For couples undergoing premarital examinations (non-pregnant women and their partners), an NGS-based universal screening strategy was employed, combining CBC with NGS.

The screening and prevention protocols followed the *Technical Service Specifications for Thalassemia Prevention and Control Pilot Program (Trial)* issued by the National Health Commission of China. All experimental procedures were performed in accordance with relevant guidelines and regulations, including the Declaration of Helsinki. All participants received pre-test counseling and provided written informed consent. The use of de-identified data for this retrospective analysis was covered by the original informed consent and approved by the Ethics Committee of Ganzhou Women and Children’s Health Care Hos (Approval No. 2024002).

Although the two screening strategies were applied to different clinical subgroups (pregnant vs. premarital couples), the study population was drawn from the same geographical region and timeframe, with participants largely overlapping in age (predominantly 20–40 years). Moreover, these two subgroups are not mutually exclusive in clinical practice, as individuals undergoing premarital screening may subsequently become pregnant and enter the prenatal screening pathway, and vice versa. This dynamic conversion further supports the comparability of the two cohorts. The large sample size (*N* > 90,000) further helps mitigate potential selection bias and enhances the reliability of the inter-strategy comparison.

### Traditional three-step cascade screening protocol

For pregnant women attending prenatal examinations and their spouses, 2 mL of peripheral blood was collected into EDTA anticoagulant tubes.

Blood cell analysis was performed using a Mindray BC-7500 automated hematology analyzer (Mindray, Shenzhen, China). A positive result was defined as a mean corpuscular volume (MCV) < 82 fL and/or a mean corpuscular hemoglobin (MCH) < 27 pg.

Samples from subjects with positive blood cell analysis results, along with those of their spouses, were analyzed using capillary electrophoresis on a Capillarys 2 Flex Piercing system (Sebia, France). The system interprets results based on hemoglobin migration rates to identify and quantify hemoglobin variants. A positive electrophoresis result was defined as HbA2 < 2.6% or HbA2 > 3.5%, or the presence of abnormal hemoglobin bands (e.g., Hb H, Hb Bart’s).

When both the pregnant woman and her spouse tested positive in HE, their samples were sent to Ganzhou Women and Children’s Health Care Hos for thalassemia gene detection using the PCR-based flow hybridization method.

DNA was extracted from 200 µL of each sample using an automated procedure on an NP968-S nucleic acid extractor (Tianlong, Xi’an, China) according to the manufacturer’s instructions. The concentration and purity of the extracted DNA were determined using a NanoDrop microspectrophotometer (Thermo Fisher Scientific, USA). Subsequently, 5 µL of sample DNA was added to 45 µL of reaction mix in a PCR tube, and amplification was performed on an ABI 7300 thermal cycler (Applied Biosystems, USA). The PCR products were denatured at 95 °C and hybridized on an HB-2012 A hybridization instrument (Hybribio Biotechnology, Guangzhou, China). This assay detects three non-deletional α-thalassemia point mutations (*HBA2*:c.369C>G, *HBA2*:c.427T>C, *HBA2*:c.377T>C), three deletional α-thalassemia variants (--^SEA^, -α^3.7^, -α^4.2^), and 17 β-thalassemia point mutations commonly found in China. In this manuscript, large deletions are designated by conventional names (e.g., --^SEA^), whereas point mutations are described using HGVS nomenclature. A full correspondence between the conventional and HGVS names is provided in Supplementary Table 1.

### NGS-based universal screening protocol

 For couples undergoing premarital examinations, 2 mL of peripheral blood was collected from each individual into two separate EDTA anticoagulant tubes.

One EDTA tube was used for blood cell analysis on a Mindray BC-7500 automated hematology analyzer.

The second EDTA tube was sent directly to Ganzhou Women and Children’s Health Care Hos for genetic testing. Genomic DNA was extracted from 200 µL of whole blood using the QIAamp DNA Blood Mini Kit (Qiagen, Hilden, Germany). DNA was arrayed in 96-well plates, and concentration was quantified using a Qubit 3.0 fluorometer (Thermo Fisher Scientific, Waltham, MA, USA). The DNA fragments of the *HBA1*, *HBA2*, and *HBB* genes were amplified by PCR and then subjected to ultrasonic fragmentation to obtain segments of approximately 200 base pairs. Following end-repair, sequencing adapters were ligated to construct the sequencing library. Sequencing was performed on an MGISEQ-200 platform (MGI, Shenzhen, China) in paired-end (PE100) mode. Finally, sequencing data were analyzed using the Halos system to identify mutation sites in the target genes. The detection scope covers four common α-thalassemia deletions (--^SEA^, -α^3.7^, -α^4.2^, --^THAI^), three common β-thalassemia deletions (Chinese (^A^γδβ)^0^, SEA-HPFH, Taiwanese deletion), as well as point mutations in the *HBA1*, *HBA2*, and *HBB* genes, as previously described^19^.

### Clinical severity assessment

Upon receiving genetic test results, a clinical severity assessment was performed for couples in which both partners were identified as thalassemia carriers. Based on the potential genotypes and corresponding clinical severity expected in their offspring, these dual-positive couples were stratified into eight specific severity categories:

1. Different type (one partner with α-thalassemia and the other with β-thalassemia; offspring typically asymptomatic or mild);
2. α-high severity (at risk for homozygous α-thalassemia major, a lethal condition; prenatal diagnosis strongly recommended);
3. α-intermediate-high severity (at risk for severe non-deletional Hb H disease, e.g., --^SEA^/α^*HBA2*:c.427T>C^α; often transfusion-dependent; prenatal diagnosis strongly recommended);
4. α-intermediate severity (at risk for deletional Hb H disease, e.g., --^SEA^/-α^3.7^; variable but often symptomatic; prenatal diagnosis recommended);
5. α-intermediate-low severity (at risk for mild non-deletional Hb H disease, e.g., --^SEA^/α^*HBA2*:c.369C>G^α; usually mild or asymptomatic; prenatal diagnosis generally not recommended);
6. α-low severity (at risk for mild α-thalassemia trait; asymptomatic; prenatal diagnosis not recommended);
7. β-high severity (at risk for β-thalassemia major, e.g., β^0^/β^+^; transfusion-dependent; prenatal diagnosis strongly recommended);
8. β-low severity (at risk for mild β-thalassemia, e.g., β^0/+^/β^++^; usually mild or asymptomatic; prenatal diagnosis not recommended).

This detailed stratification facilitated a precise analysis of detection efficacy across clinically distinct severity groups. Notably, categories 2, 3, 4, and 7 (α-high, α-intermediate-high, α-intermediate, and β-high severity) are considered clinically actionable and warrant prenatal diagnosis and genetic counseling, whereas categories 1, 5, 6, and 8 generally do not require invasive prenatal interventions.

### Statistical analysis

Statistical analysis was performed using SPSS software (version 27.0). Continuous data are expressed as mean ± standard deviation (SD), and categorical data are expressed as counts and percentages (%). Between-group comparisons of categorical variables were conducted using the chi-square test, and residual analysis was employed to identify the specific genotypes responsible for the differences between the screening strategies. A *p*-value of < 0.05 was considered statistically significant.

## Results

### Detection rates

NGS-based universal screening: Among 54,357 participants, the total thalassemia detection rate was 15.05%, including α-thalassemia (10.99%), β-thalassemia (3.54%), and α-compound β-thalassemia (0.52%). (Table 1).

**Table 1:** Comparison of Thalassemia Detection Rates between NGS-based Universal Screening and Traditional Cascade Screening.

Screening parameter	NGS-based universal screening (*n* = 54,357)	Traditional cascade screening (*n* = 37,500)	Ratio (NGS/Traditional)
Total Thalassemia Carriers	8,183 (15.05%)	1,191 (3.18%)*	4.74
α-thalassemia	5,976 (10.99%)	794 (2.12%)	5.19
β-thalassemia	1,925 (3.54%)	352 (0.94%)	3.78
α-compound β-thalassemia	282 (0.52%)	46 (0.12%)	4.23
Subjects proceeding to hemoglobin electrophoresis	0 (0%)	9690 (25.84%)	—
Subjects proceeding to genetic testing	54,357 (100.0%)	2,332 (6.22%)	—
Positive rate among genetically tested	15.05%	1,191/2,332 (51.07%)	—

*The overall detection rate (3.18%) for the traditional group was calculated based on the total screened population (*n* = 37,500). NGS, Next-generation sequencing.

Traditional cascade screening: Among the 37,500 subjects examined, 4,845 couples were tested for HE due to positive results in at least one CBC. Of these, 1,170 couples tested positive for both HE markers. A total of 2,332 individuals from these 1,170 couples underwent genetic testing, resulting in a genetic testing rate of 6.22%. Among the individuals who underwent genetic testing, 1,141 tested negative and 1,191 tested positive, yielding a positive rate of 51.07% (detection rate: 3.18%). The detected conditions included α-thalassemia in 794 cases (detection rate: 2.12%), β-thalassemia in 352 cases (detection rate: 0.94%), and α-compound β-thalassemia in 46 cases (detection rate: 0.12%). (Table 1).

The detection rates for all thalassemia types were significantly higher in the NGS group (Table 1). Specifically, the overall carrier detection rate with NGS-based universal screening was 4.74 times that of the traditional cascade screening method. Similarly, the detection rates for α-thalassemia, β-thalassemia, and α-compound β-thalassemia were 5.19 times, 3.78 times, and 4.23 times higher, respectively, with the NGS-based strategy.

### Regional distribution of carrier rates

**Fig. 1:**
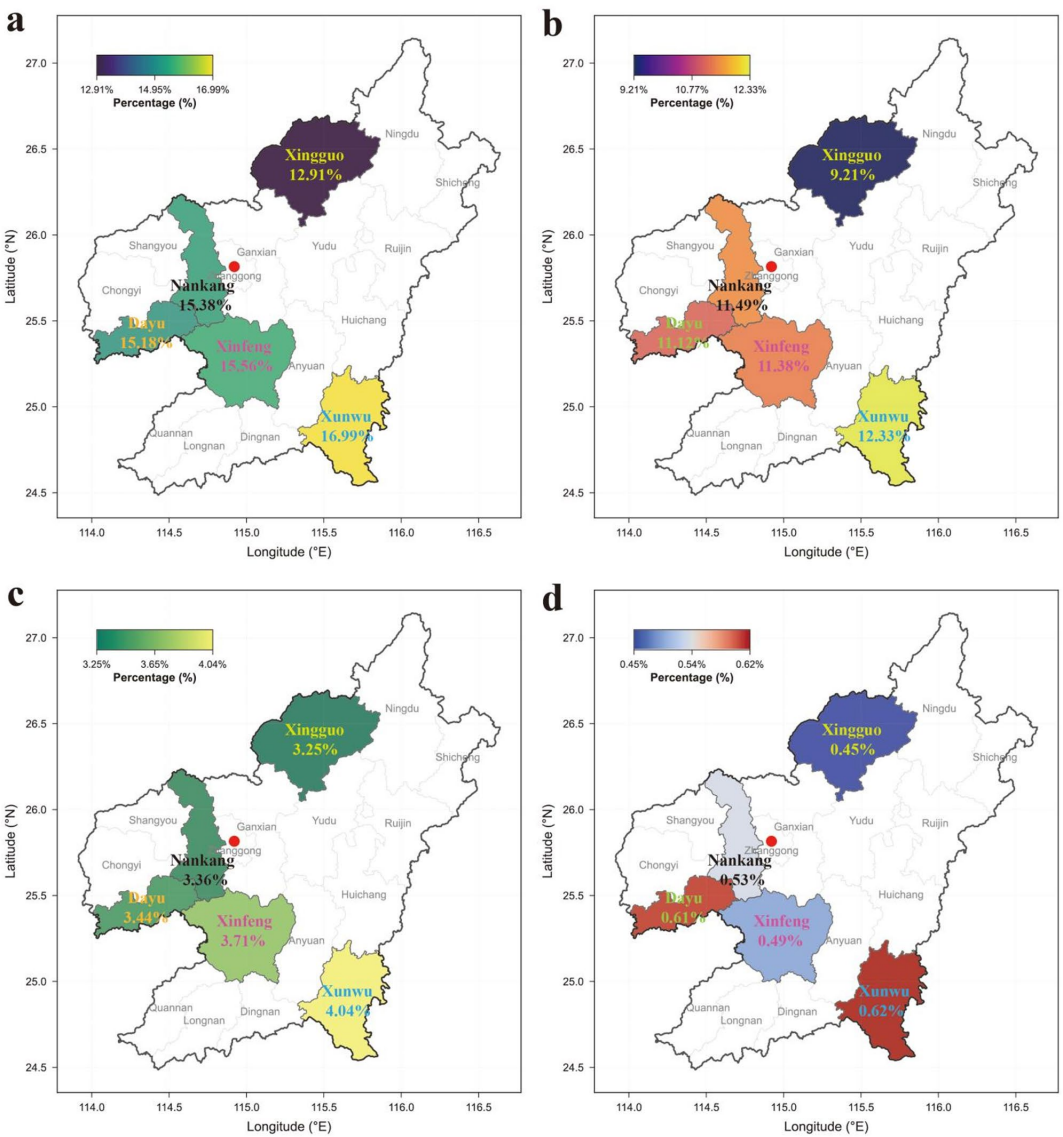
Distribution of thalassemia carrier rates across five target counties. (**a**) Overall thalassemia carrier rate. (**b**) α-thalassemia carrier rate. (**c**) β-thalassemia carrier rate. (**d**) α-compound β-thalassemia carrier rate.

Due to the substantially lower detection rates of the traditional method, which inadequately reflect the true prevalence, the NGS-based data were utilized to analyze regional disparities across the five counties. The overall thalassemia carrier rates and the rates for each subtype (α-thalassemia, β-thalassemia, and α-compound β-thalassemia) are presented geographically in Fig. 1.

In brief, the overall carrier rates were 15.56%, 15.38%, 12.91%, 16.99%, and 15.18% for Xinfeng, Nankang, Xingguo, Xunwu, and Dayu counties, respectively (Fig. 1a). A similar geographical pattern was observed for the subtypes: α-thalassemia rates were 11.38%, 11.49%, 9.21%, 12.33%, and 11.12% (Fig. 1b); β-thalassemia rates were 3.71%, 3.36%, 3.25%, 4.04%, and 3.44% (Fig. 1c); and α-compound β-thalassemia rates were 0.49%, 0.53%, 0.45%, 0.62%, and 0.61% (Fig. 1d).

Overall, the data visualized in Fig. 1 indicate a trend of higher thalassemia carrier rates in the eastern and southern regions compared to the western and northern parts of the studied area.

### Genotypic profiles of carriers

Among α-thalassemia carriers (α-thalassemia and α-compound β-thalassemia) detected by NGS-based universal screening, the --^SEA^/αα genotype was the most prevalent, accounting for 53.52% (3349/6258). Other frequent genotypes included -α^3.7^/αα, -α^4.2^/αα, α^*HBA2*:c.369C>G^α/αα, α^*HBA2*:c.427T>C^α/αα, and α^*HBA2*:c.377T>C^α/αα, with proportions of 28.08%, 8.45%, 3.99%, 2.14%, and 0.88%, respectively. Collectively, these six genotypes comprised 97.06% (6074/6258) of all α-thalassemia cases identified by NGS (Supplementary Table 2). In the traditional cascade screening group, --^SEA^/αα was also the most common genotype but constituted a significantly higher proportion of 73.81% (620/840). The other prevalent genotypes were -α^3.7^/αα (15.48%), -α^4.2^/αα (4.05%), α^*HBA2*:c.427T>C^α/αα (2.50%), --^SEA^/-α^3.7^ (1.55%), and α^*HBA2*:c.369C>G^α/αα (1.31%), together representing 98.69% of α-thalassemia cases in this group (Supplementary Table 3).

Among β-thalassemia carriers detected (β-thalassemia and α-compound β-thalassemia) by NGS-based universal screening, the most common genotypes were β^*HBB*:c.316–197C>T^/β^N^ (37.65%), β^*HBB*:c.126_129delCTTT^/β^N^ (28.50%), β^*HBB*:c.-78A>G^/β^N^ (12.01%), β^*HBB*:c.52A>T^/β^N^ (5.80%), β^*HBB*:c.-11_-8delAAAC^/β^N^ (4.44%), and β^*HBB*:c.-100G>A^/β^N^ (2.54%). These six genotypes constituted 90.94% (2007/2207) of all β-thalassemia cases (Supplementary Table 4). In the traditional cascade screening group, the top genotypes were β^*HBB*:c.316–197C>T^/β^N^ (37.69%), β^*HBB*:c.126_129delCTTT^/β^N^ (29.90%), β^*HBB*:c.-78A>G^/β^N^ (13.07%), β^*HBB*:c.52A>T^/β^N^ (10.30%), β^*HBB*:c.85dupC^/β^N^ (2.76%), and β^*HBB*:c.217dupA^/β^N^ (1.51%), together accounting for 95.23% (379/398) of cases (Supplementary Table 5).

A comparative analysis of genotype distribution patterns and detection biases was conducted for the major α- and β-thalassemia genotypes. Data from both screening strategies were analyzed after consolidating genotypes with low counts into an “other” category, resulting in nine distinct genotype classifications for each type. For α-thalassemia, these were: --^SEA^/αα, -α^3.7^/αα, -α^4.2^/αα, α^*HBA2*:c.369C>G^α/αα, α^*HBA2*:c.427T>C^α/αα, α^*HBA2*:c.377T>C^α/αα, --^SEA^/-α^3.7^, --^SEA^/-α^4.2^, and other. For β-thalassemia, the classifications were: β^*HBB*:c.316–197C>T^/β^N^, β^*HBB*:c.126_129delCTTT^/β^N^, β^*HBB*:c.-78A>G^/β^N^, β^*HBB*:c.52A>T^/β^N^, β^*HBB*:c.-11_-8delAAAC^/β^N^, β^*HBB*:c.-100G>A^/β^N^, β^*HBB*:c.85dupC^/β^N^, β^*HBB*:c.79G>A^/β^N^, and other.

**Fig. 2:**
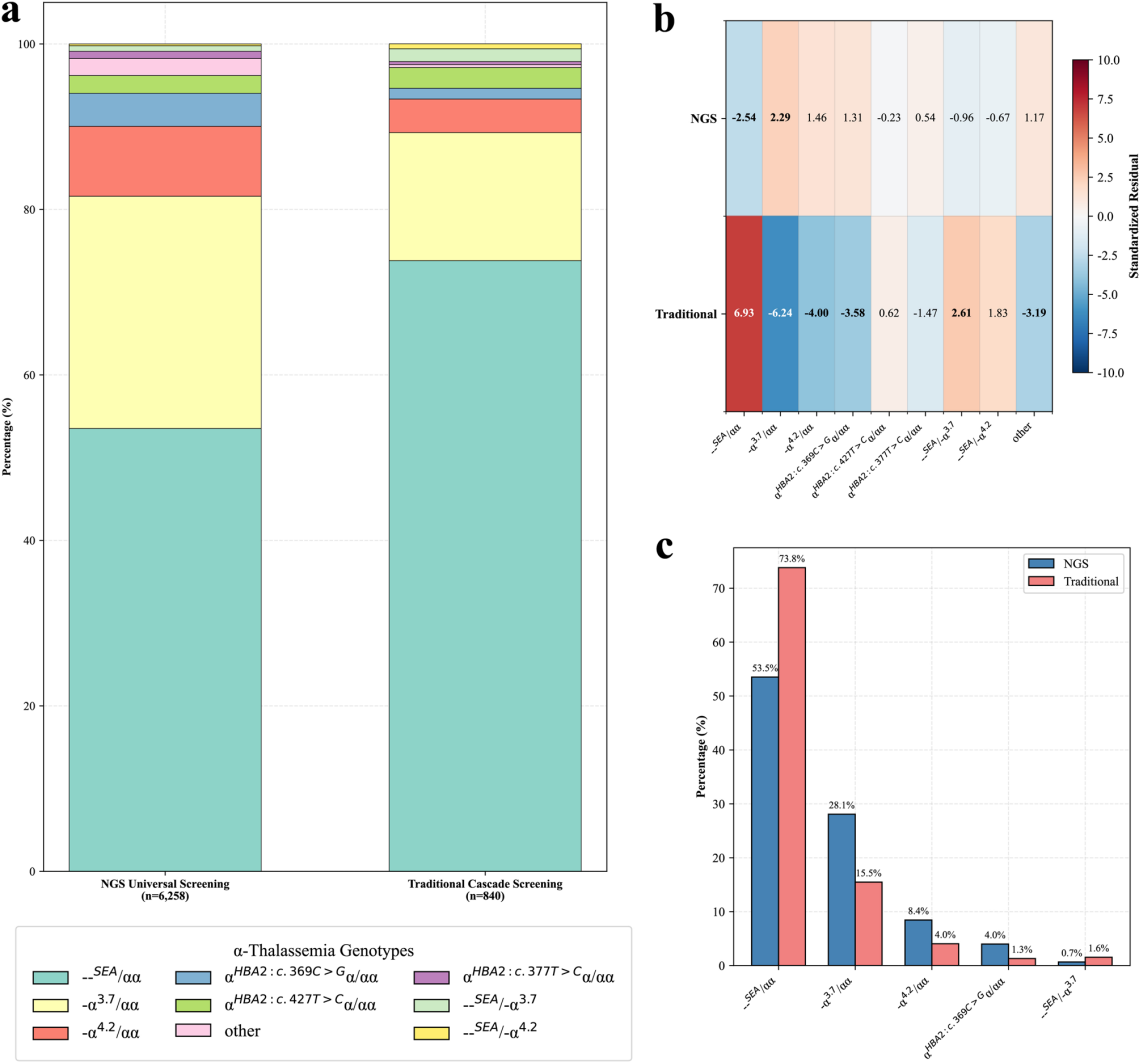
Comparison of α-thalassemia genotype distribution between NGS-based universal screening and traditional cascade screening. (**a**) Stacked bar chart of genotype distribution. (**b**) Standardized residuals heatmap. (**c**) Significantly different genotypes between two strategies.

Pearson’s chi-square test revealed a statistically significant difference in α-thalassemia genotype distribution between the two screening strategies (χ² = 157.46, df = 8, *P* < 0.001) (**Supplementary Table 6**). Analysis of standardized residuals identified five genotypes with significant detection bias (|standardized residual| > 1.96). Compared to NGS-based universal screening, traditional cascade screening demonstrated substantial under-representation of genotypes typically associated with silent phenotypes, including -α^3.7^/αα, -α^4.2^/αα, and α^*HBA2*:c.369C>G^α/αα. In contrast, genotypes with more pronounced hematological manifestations, specifically the mild form --^SEA^/αα and intermediate forms such as --^SEA^/-α^3.7^, were significantly over-represented in the traditional cascade screening cohort (Fig. 2).

**Fig. 3:**
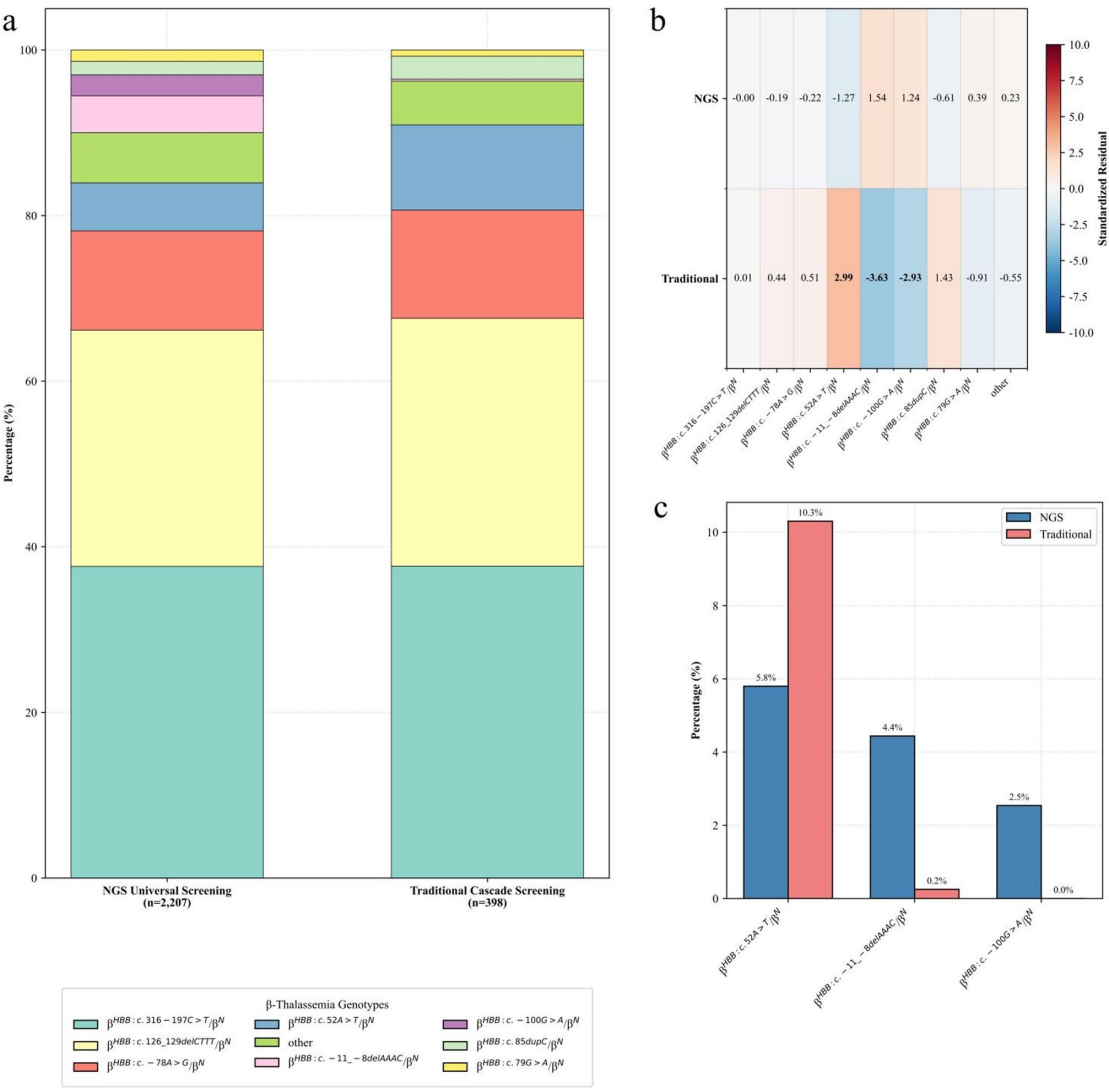
Comparison of β-thalassemia genotype distribution between NGS-based universal screening and traditional cascade screening. (**a**) Stacked bar chart of genotype distribution. (**b**) Standardized residuals heatmap. (**c**) Significantly different genotypes between two strategies.

Similarly, the genotype distribution of β-thalassemia also differed significantly between the two strategies (χ² = 40.47, df = 8, *P* < 0.001) (Supplementary Table 7). Interestingly, in contrast to α-thalassemia, the three most prevalent β-thalassemia genotypes, collectively accounting for approximately 80% of cases, showed remarkably consistent proportional distributions across both screening approaches: β^*HBB*:c.316–197C>T^/β^N^ (37.65% vs. 37.69%), β^*HBB*:c.126_129delCTTT^/β^N^ (28.50% vs. 29.90%), and β^*HBB*:c.−78A>G^/β^N^ (12.01% vs. 13.07%). Standardized residuals analysis revealed that the primary source of the overall distributional difference stemmed from two β⁺⁺ genotypes—β^*HBB*:c.−11_−8delAAAC^/β^N^ and β^*HBB*:c.−100G> A^/β^N^—along with β^*HBB*:c.52A>T^/β^N^. Notably, the two β⁺⁺ genotypes collectively accounted for 6.98% (4.44% + 2.54%) of cases in the NGS-based universal screening but only 0.25% (0.25% + 0%) in traditional cascade screening, representing a 27.77-fold difference in proportional composition. This substantial under-detection of the β⁺⁺ genotypes in the traditional screening concurrently led to a relative over-representation of β^*HBB*:c.52A>T^/β^N^ in that cohort (Fig. 3).

### Identification of at-risk couples and screening implications

Following genetic testing, spousal genotype matching was performed to assess the risk of couples having offspring with thalassemia. In the NGS-based universal screening cohort, 27,211 couples were successfully matched. Among these, 19,374 (71.2%) were double-negative, 7160 (26.3%) were single-positive, and 677 (2.49%) were double-positive. In the traditional cascade screening cohort, of the 1162 couples who proceeded to genetic testing following the screening cascade, 234 (20.1%) were double-negative, 669 (57.6%) were single-positive, and 259 (22.3%) were double-positive. When calculated based on all 18,750 couples in the traditional cascade screening cohort, the double-positive rate was 1.38%, significantly lower than the NGS-based universal screening cohort’s 2.49%, yielding a relative detection rate of 55.4% compared to NGS.

**Fig. 4:**
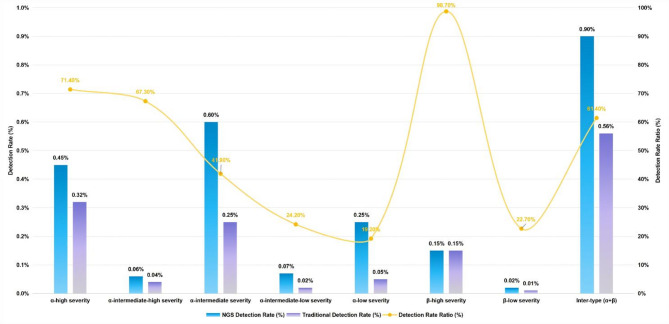
Comparison of detection rates across different clinical severity categories.

The detection rates for couples across specific thalassemia severity categories are detailed in Supplementary Tables 8 and visually compared in Fig. 4. A consistent pattern of lower detection was observed for all α-thalassemia-related severity categories in the traditional cascade screening cohort, as reflected in the detection rate ratios. For instance, the detection rate for α-high severity couples (at risk for Bart’s hydrops fetalis) showed a detection rate ratio of 71.4% (0.320% vs. 0.448%). The ratio was 41.9% for α-intermediate severity couples (0.251% vs. 0.599%). The most pronounced difference was in the α-low severity category, with a detection rate ratio of only 19.2% (0.048% vs. 0.250%).

In contrast, the detection rates for β-high severity couples were nearly identical, yielding a detection rate ratio of 98.7% (0.149% vs. 0.151%). For couples at risk for both α- and β-thalassemia (inter-type), the detection rate ratio was 61.4% (0.555% vs. 0.904%).

### Testing costs and turnaround time

Based on unit costs (CBC: USD 1; HE: USD 4; NGS: USD 20; PCR-based flow hybridization: USD 30), we calculated total and per-capita costs for both strategies.

For the NGS-based universal screening strategy applied to 54,357 individuals, the total cost was USD 1,141,497 (USD 21 per person × 54,357), yielding a per-capita cost of USD 21. For the traditional cascade screening strategy applied to 37,500 participants, the total cost was USD 146,220, derived as follows: [(USD 1 × 37,500 for CBC) + (4,845 couples × 2 tests × USD 4 for HE) + (2,332 tests × USD 30 for PCR-based flow hybridization)]. This resulted in a per-capita cost of USD 3.90. Consequently, the per-capita cost of the traditional strategy was only 18.57% of that for the NGS-based strategy (Table 2).

**Table 2:** Cost and timeline comparison between NGS-based universal screening and traditional cascade screening strategies.

Parameter	NGS-based Universal Screening	Traditional Cascade Screening
Screened Population	54,357	37,500
Complete Blood Count	54,357	37,500
Hemoglobin Electrophoresis	–	9,690 (4,845 × 2)
Next-Generation Sequencing	54,357	–
PCR-based flow hybridization	–	2,332
Total Cost (USD)	1,141,497	146,220
Per-capita Cost (USD)	21	3.9
Per-capita Cost Ratio (Traditional/NGS)	100%	18.57%
Mean Turnaround Time (days)	13.06 ± 3.06	6.40 ± 3.43
Turnaround Time Range (days)	7–62	2–41

Regarding the testing timeline, the average turnaround time was 13.06 ± 3.06 days (range: 7–62 days) for the NGS-based strategy, compared to 6.40 ± 3.43 days (range: 2–41 days) for the traditional strategy.

## Discussion

This study presents a comparative analysis of population data from a high-prevalence Hakka region in southern China. To our knowledge, it represents the first direct comparison within the same cohort that systematically evaluates the comprehensive performance of an NGS-based universal screening strategy against the traditional three-step cascade approach for thalassemia prevention. Beyond quantifying differences in detection rates, this research elucidates the underlying mechanisms and real-world impact of each screening paradigm across multiple dimensions: genotype profiles, identification of at-risk couples, clinical implications, and cost-effectiveness. These findings offer direct evidence to inform and optimize prevention strategies in high-prevalence settings.

We identified a marked discrepancy in detection rates, with NGS-based universal screening yielding a carrier detection rate approximately 4.74 times higher than the traditional cascade method. Historically, due to the high cost of genetic testing, most studies derived their data from individuals who tested positive on CBC or HE, leading to reported local carrier rates as high as 16.39%-73%—an overestimation likely resulting from selection bias^20–22^. In the present study, the traditional cascade screening strategy reduced the genetic testing rate to 6.22% through stringent sequential screening; however, the positivity rate among those tested reached 51.07%, which far exceeds the rate observed in the NGS-based universal screening cohort where all individuals underwent screening. Conversely, studies relying on data from traditional screening that calculated carrier rates by dividing genetic positives by all screened cases would substantially underestimate the true local carrier rate^23,24^. Thus, previous studies may not accurately reflect the actual thalassemia carrier prevalence in this region.

Although some universal screening approaches can mitigate initial screening bias, limitations such as insufficient sample size or detection methods restricted to common variants may still introduce inaccuracies in estimating local carrier rates^25^. In this study, NGS was applied to a large cohort of over 50,000 reproductive-age individuals. The NGS method demonstrates strong detection performance for most common deletional thalassemias and nearly all point mutations^26^. With an accuracy of 98.48% as validated by third-generation sequencing, despite certain limitations such as detecting α-globin gene duplications, rare deletions, and complex rearrangements, the carrier rate derived from NGS-based universal screening in this study is likely representative of the true local prevalence.

Significant variations in carrier rates were observed even among the five counties within our study, generally showing higher rates in the south compared to the north. Moreover, the genotypic profile in our cohort differs from those reported in other southern regions of China, with distinct predominant genotypes. For α-thalassemia, the --^SEA^ deletion is most prevalent in our region, whereas the -α^3.7^ deletion is particularly common in the Dai ethnic group of Yunnan Province, China, as well as in many African countries^27,28^. For β-thalassemia, the *HBB*:c.126_129delCTTT mutation is most common in Guangxi and Guangdong, while *HBB*:c.316–197C>T predominates in our region^29^. Meizhou, a city bordering Ganzhou, shares a predominantly Hakka population similar to that of Ganzhou. The ranking and proportion of the main α- and β-thalassemia genotypes reported in Meizhou align closely with the findings from the traditional cascade screening cohort in our study, yet differ from other regions, highlighting the distinct genetic profile of thalassemia in the Hakka population^30^. These differences underscore the profound influence of geographical isolation and population genetics on thalassemia carrier prevalence and genotype distribution, emphasizing the necessity of population-specific genetic databases.

Beyond the substantial difference in detection rates, the two screening strategies also exhibited marked disparities in the proportional distribution of major genotypes. For α-thalassemia, traditional cascade screening demonstrated significant detection bias, favoring genotypes associated with more pronounced hematological phenotypes (e.g., --^SEA^/αα and --^SEA^/-α^3.7^), while consistently under-detecting silent carriers, including -α^3.7^/αα, -α^4.2^/αα, and α^*HBA2*:c.369C>G^α/αα. For β-thalassemia, the distribution of the predominant genotypes was largely similar between the two strategies. The primary discrepancy stemmed from the severe under-detection of β⁺⁺ genotypes, specifically *HBB*:c.-11_-8delAAAC and *HBB*:c.-100G>A, in the traditional cascade screening cohort. This systematic bias suggests that previous estimates relying on traditional screening methods may have substantially underestimated the true population proportions of both silent α-thalassemia carriers and β⁺⁺- thalassemia variants.

Subsequent couple risk assessment revealed severe under-detection across all α-thalassemia-related severity categories in the traditional cascade screening strategy, exhibiting a “funnel effect” where under-detection worsened for lower-severity categories. Crucially, even for α-high-severity couples, the under-detection rate was 28.6%. In stark contrast, detection for β-high severity couples was nearly equivalent (detection rate ratio: 98.7%). This performance mismatch had direct clinical consequences: follow-up revealed at least three pregnancies in the traditional cascade screening cohort that progressed to Hb Bart’s hydrops fetalis, diagnosed only late in gestation due to fetal hydrops, precluding timely intervention and causing significant harm. Additionally, two infants with non-deletional Hb H disease were born, requiring regular transfusion.

Although heterozygous α⁺-thalassemia carriers have a mild phenotype, their combination with α⁰-thalassemia can result in Hb H disease. Homozygous α⁰-thalassemia (--^SEA^/--^SEA^) is the most severe form, leading to hydrops fetalis^31^. However, Hb H disease, particularly the non-deletional form (e.g., --^SEA^/α^*HBA2*:c.427T>C^α), causes moderate to severe hemolytic anemia requiring regular blood transfusions, typically every 1–2 months^32^. In our region, more than 100 children with Hb H disease require regular transfusions, accounting for approximately one-quarter of all transfusion-dependent thalassemia children, rather than β-thalassemia major. This imposes a substantial burden on local thalassemia prevention and control programs. Therefore, detecting α⁺-thalassemia carriers (e.g., -α^3.7^/αα, -α^4.2^/αα, α^*HBA2*:c.427T>C^α/αα) is clinically meaningful for identifying couples at risk for Hb H disease (--^SEA^/α⁺), particularly in regions with high α⁰-thalassemia prevalence like ours. The systematic under-detection of these carriers by the traditional algorithm has direct consequences for prevention programs.

This disparity may be attributed to the epidemiological context for which the traditional stepwise screening algorithm was originally designed. The protocol was first implemented in Mediterranean and European countries, where β-thalassemia is the predominant form, and in African regions, where hemoglobinopathies caused by β-globin mutations present with distinctive electrophoretic patterns easily captured by HE^33–35^. In contrast, across East and Southeast Asia, including our region, the carrier rate for α-thalassemia significantly exceeds that of β-thalassemia^36^. Although the hematological phenotype of the --^SEA^/αα genotype is typically evident, a subset of individuals may exhibit an Hb A2 level within the normal range on HE. The data from this study corroborate this mismatch: while the traditional cascade screening strategy performs well for β-high-severity detection, its performance for α-thalassemia is suboptimal. These findings underscore that a one-size-fits-all thalassemia screening protocol requires adaptation to local epidemiological and genetic landscapes. For regions like ours with a high α-thalassemia burden, modifying the strict sequential three-step cascade to a parallel screening model—whereby genetic testing is initiated if either CBC or HE is positive—may represent a more effective and appropriate prevention strategy. Notably, this modified approach has been implemented in our region since 2024 (after the study period), and preliminary observations suggest a marked reduction in the under-detection rate of α-thalassemia.

Compared with the traditional cascade screening strategy, the NGS-based universal screening approach significantly reduces the risk of under-detection inherent in the cascade model. Additionally, it enables the identification of rare deletional forms and point mutations that are typically missed by conventional methods. Currently, most genetic testing kits used in China, including the traditional PCR-based flow hybridization assay employed in this study, are commercially approved kits (certified by the National Medical Products Administration) that target mutations considered “common” nationwide^32^. Modifying such kits to include additional probes is not straightforward, as it requires regulatory re-approval, which is both costly and time-consuming. Notably, our NGS data provide evidence for identifying rare variants with relatively high carrier frequency in our region, such as the 21 --^THAI^ carriers identified in this study (0.039% of the screened population), suggesting that although this deletion is rare nationally, it may warrant inclusion in region-specific PCR panels for Ganzhou or Jiangxi Province. This represents a practical benefit of NGS: informing the improvement of lower-cost targeted assays for specific populations. However, this comprehensive strategy also introduces new challenges. Certain variants—such as *HBA1*:c.354_355insATC, *HBB*:c.−11_−8delAAAC, and *HBB*:c.-100G>A—may be misclassified as pathogenic in the absence of population-specific databases, potentially leading to unnecessary prenatal diagnoses and interventions^37,38^. For example, accumulating evidence has subsequently reclassified the *HBB*:c.-100G>A variant as benign^39^. Consequently, the genetic counseling recommendation for couples in which one partner carries this variant and the other has β-thalassemia has shifted from advising prenatal diagnosis to not recommending it. Paradoxically, the traditional cascade screening strategy, by missing these clinically subtle variants, inadvertently avoided such unnecessary interventions.

In terms of health economics, the two strategies present a trade-off. The traditional cascade screening strategy has a clear cost-speed advantage (per-capita cost 18.57%; ~7-day shorter turnaround), making it attractive in resource-limited settings. However, this “low-cost, high-efficiency” profile comes at the significant expense of screening completeness and accuracy, posing a severe clinical risk. Conversely, the NGS-based universal screening strategy’s more complex workflow contributes to a longer reporting cycle (~ 13 days), potentially delaying prenatal diagnosis. Therefore, a direct comparison of time and monetary costs is insufficient. Any complete evaluation must weigh the procedural delays of universal screening against the more consequential risks of diagnostic delay and missed diagnoses inherent in the traditional approach.

Several limitations of this study should be acknowledged. First, the two screening strategies were applied to distinct clinical subgroups (pregnant women vs. premarital couples) rather than through a randomized controlled design. Although the large sample size and demographic overlap mitigate potential selection bias, we cannot entirely exclude the possibility of residual confounding. Second, the traditional cascade screening protocol only proceeded to genetic testing for subjects with positive initial screening results (reduced MCV/MCH), which inevitably leads to a substantially lower observed carrier rate. Consequently, a direct comparison of crude carrier rates between the two strategies may not be entirely appropriate. Third, as the findings are derived from a specific Hakka population in southern China, caution is warranted when extrapolating these results to other ethnic groups or regions with different genetic backgrounds and healthcare infrastructures.

## Conclusion

This large-scale comparative study demonstrates that while traditional cascade screening offers advantages in cost and speed, it suffers from systematic under-detection, particularly for α-thalassemia. Beyond the 4.74‑fold higher carrier detection rate of NGS, the traditional strategy missed 28.6% of α‑high‑severity couples and 32.7% of α‑intermediate‑high‑severity couples—deficits that directly translate into preventable cases of Hb Bart’s hydrops fetalis and transfusion‑dependent Hb H disease. NGS‑based universal screening provides a far more accurate assessment of the true thalassemia burden and genotype diversity in this high‑prevalence Hakka population. For regions with a high α‑thalassemia burden, modifying the strict sequential three‑step cascade to a parallel screening model (initiating genetic testing based on either low MCV/MCH or abnormal hemoglobin electrophoresis) may substantially improve detection. These findings underscore that screening efficacy is profoundly influenced by local epidemiology, and population‑specific databases are essential for optimizing prevention strategies in high‑prevalence areas.

## Supplementary Information

Below is the link to the electronic supplementary material.

Supplementary Material 1

Supplementary Material 2

## Data Availability

All pertinent data for this study are provided in text, figures, and in the supplementary files. Further information is available from the corresponding author upon request.
