# Supplementary material for: Comparing traditional and NGS-based screening strategies for thalassemia in a high-prevalence Hakka population: a population-based study
Source: Sci Rep. 2026 Aug 18;16:25882. doi: 10.1038/s41598-026-53002-8 (PMC13486634; doi:10.1038/s41598-026-53002-8)
Supplement: Supplementary file 1 [file 41598_2026_53002_MOESM1_ESM.docx]

Supplementary material

**Supplementary Figure 1.** Basic Geographic Information and Distribution of Target Study Areas in Ganzhou.

**Supplementary Table 1.** Correspondence between conventional and HGVS nomenclature for thalassemia mutations detected in this study.

| **Conventional Name** | **HGVS Name** | **Phenotype / Type** |
| --- | --- | --- |
| --^SEA^ | NC_000016.10:g.165401_184701del | α^0^-thalassemia |
| -α^3.7^ | NG_000006.1:g.34247_38050del | α^+^-thalassemia |
| -α^4.2^ | NC_000016.10:g.169818_174075del | α^+^-thalassemia |
| --^THAI^ | NC_000016.10:g.149863_183312del | α^0^-thalassemia |
| Chinese (^A^γδβ)^0^ | NC_000011.10:g.5169918_5248821del | ^G^γ(^A^γδβ)^0^ thalassemia |
| SEA-HPFH | NC_000011.10:g.5201647_5229059del | β^N/A^-thalassemia |
| Taiwanese deletion | NG_000007.3:g.69997_71353del1357 | β^N/A^-thalassemia |

**Supplementary Table 2.** Genotype distribution and detection rates of α-thalassemia carriers identified by NGS-based universal screening.

| **Genotypes** | **Phenotype** | **Cases** | **Constituent ratio (%)** | **Detection rate (%)*** |
| --- | --- | --- | --- | --- |
| αα/--^SEA^ | α^0^/α | 3349 | 53.52% | 6.16% |
| αα/-α^3.7^ | α^+^/α | 1757 | 28.08% | 3.23% |
| αα/-α^4.2^ | α^+^/α | 529 | 8.45% | 0.97% |
| α^HBA2:c.369C>G^α/αα | α^N/A^/α | 250 | 3.99% | 0.46% |
| α^HBA2:c.427T>C^α/αα | α^+^/α | 134 | 2.14% | 0.25% |
| α^HBA2:c.377T>C^α/αα | α^+^/α | 55 | 0.88% | 0.10% |
| -α^3.7^/--^SEA^ | α^+^/α^0^ | 41 | 0.66% | 0.08% |
| α^HBA1:c.354_355insATC^α/αα | α^+^/α | 22 | 0.35% | 0.04% |
| -α^3.7^/-α^3.7^ | α^+^/α^+^ | 20 | 0.32% | 0.04% |
| αα/--^THAI^ | α^0^/α | 17 | 0.27% | 0.03% |
| -α^4.2^/--^SEA^ | α^+^/α^0^ | 14 | 0.22% | 0.03% |
| α^HBA2:c.369C>G^α/--^SEA^ | α^N/A^/α^0^ | 11 | 0.18% | 0.02% |
| α^HBA2:c.91_93delGAG^α/αα | α^+^/α | 9 | 0.14% | 0.02% |
| -α^3.7^/-α^4.2^ | α^+^/α^+^ | 7 | 0.11% | 0.01% |
| Hkαα/--^SEA^ | α^0^/α | 5 | 0.08% | 0.01% |
| α^HBA2:c.427T>C^α/--^SEA^ | α^+^/α^0^ | 5 | 0.08% | 0.01% |
| α^HBA1:c.354_355insATC^α/--^SEA^ | α^+^/α^0^ | 4 | 0.06% | 0.01% |
| α^HBA2:c.2delT^α/αα | α^+^/α | 4 | 0.06% | 0.01% |
| -α^4.2^/-α^4.2^ | α^+^/α^+^ | 3 | 0.05% | 0.01% |
| -α^4.2^/--^THAI^ | α^+^/α^0^ | 3 | 0.05% | 0.01% |
| Fusion gene/αα | α^+^/α | 3 | 0.05% | 0.01% |
| α^HBA2:c.377T>C^α/--^SEA^ | α^+^/α^0^ | 2 | 0.03% | 0.00% |
| α^HBA2:c.369C>G^α/-α^3.7^ | α^N/A^/α^+^ | 2 | 0.03% | 0.00% |
| α^HBA2:c.427T>C^α/-α^3.7^ | α^+^/α^+^ | 2 | 0.03% | 0.00% |
| α^HBA2:c.52G>T^α/αα | α^N/A^/α | 1 | 0.02% | 0.00% |
| α^HBA2:c.326C>A^α/αα | α^+^/α | 1 | 0.02% | 0.00% |
| α^HBA2:c.427T>C^α/-α^4.2^ | α^+^/α^+^ | 1 | 0.02% | 0.00% |
| α^HBA2:c.369C>G^α/-α^4.2^ | α^N/A^/α^+^ | 1 | 0.02% | 0.00% |
| α^HBA1:c.1A>G^α/αα | α^+^/α | 1 | 0.02% | 0.00% |
| -α^3.7^/--^THAI^ | α^+^/α^0^ | 1 | 0.02% | 0.00% |
| α^HBA2:c.184A>T^α/αα | α^+^/α | 1 | 0.02% | 0.00% |
| α^HBA1:c.354_355insATC^α/-α^3.7^ | α^+^/α^+^ | 1 | 0.02% | 0.00% |
| HKαα/-α^4.2^ | α^+^/α | 1 | 0.02% | 0.00% |
| α^HBA2:c.95+55 G>A^α/αα | α^+^/α | 1 | 0.02% | 0.00% |
| Total |  | 6258 | 100.00% | 11.51% |

*Detection rate was calculated as: (Number of cases / Total NGS-screened population [N=54,357]) × 100%.

**Supplementary Table 3.** Genotype distribution and detection rates of α-thalassemia carriers identified by traditional cascade screening.

| **Genotypes** | **Phenotype** | **Cases** | **Constituent ratio (%)** | **Detection rate (%)*** |
| --- | --- | --- | --- | --- |
| αα/--^SEA^ | α^0^/α | 620 | 53.52% | 1.65% |
| αα/-α^3.7^ | α^+^/α | 130 | 28.08% | 0.35% |
| αα/-α^4.2^ | α^+^/α | 34 | 8.45% | 0.09% |
| α^HBA2:c.427T>C^α/αα | α^+^/α | 21 | 2.14% | 0.06% |
| -α^3.7^/--^SEA^ | α^+^/α^0^ | 13 | 0.66% | 0.03% |
| α^HBA2:c.369C>G^α/αα | α^N/A^/α | 11 | 3.99% | 0.03% |
| -α^4.2^/--^SEA^ | α^+^/α^0^ | 5 | 0.22% | 0.01% |
| α^HBA2:c.377T>C^α/αα | α^+^/α | 3 | 0.88% | 0.01% |
| -α^3.7^/-α^3.7^ | α^+^/α^+^ | 1 | 0.32% | 0.00% |
| -α^4.2^/-α^4.2^ | α^+^/α^+^ | 1 | 0.05% | 0.00% |
| α^HBA2:c.427T>C^α/-α^3.7^ | α^+^/α^+^ | 1 | 0.03% | 0.00% |
| Total |  | 840 | 100.00% | 2.24% |

*Detection rate was calculated as: (Number of cases / Total traditionally-screened population [N=37,500]) × 100%.

**Supplementary Table 4.** Genotype distribution and detection rates of β-thalassemia carriers identified by NGS-based universal screening.

| **Genotypes** | **Phenotype** | **Cases** | **Constituent ratio (%)** | **Detection rate (%)*** |
| --- | --- | --- | --- | --- |
| β^HBB:c.316-197C>T^/β^N^ | β^0/+^/β^N^ | 831 | 37.65% | 1.53% |
| β^HBB:c.126_129delCTTT^/β^N^ | β^0^/β^N^ | 629 | 28.50% | 1.16% |
| β^HBB:c.-78A>G^/β^N^ | β^+^/β^N^ | 265 | 12.01% | 0.49% |
| β^HBB:c.52A>T^/β^N^ | β^0^/β^N^ | 128 | 5.80% | 0.24% |
| β^HBB:c.-11_-8delAAAC^/β^N^ | β^+^/β^N^ | 98 | 4.44% | 0.18% |
| β^HBB:c.-100G>A^/β^N^ | β^Unclear^/β^N^ | 56 | 2.54% | 0.10% |
| β^HBB:c.85dupC^/β^N^ | β^0^/β^N^ | 36 | 1.63% | 0.07% |
| Chinese (^A^γδβ)^0^/β^N^ | ^G^γ(^A^γδβ)^0^/β^N^ | 33 | 1.50% | 0.06% |
| β^HBB:c.79G>A^/β^N^ | β^+^/β^N^ | 30 | 1.36% | 0.06% |
| β^HBB:c.217dupA^/β^N^ | β^0^/β^N^ | 25 | 1.13% | 0.05% |
| SEA-HPFH/β^N^ | N/A | 12 | 0.54% | 0.02% |
| β^HBB:c.-43C>T^/β^N^ | β^++ (silent)^/β^N^ | 11 | 0.50% | 0.02% |
| β^HBB:c.45dupG^/β^N^ | β^0^/β^N^ | 9 | 0.41% | 0.02% |
| β^HHBB:c.-140C>T^/β^N^ | β^+^/β^N^ | 9 | 0.41% | 0.02% |
| β^HBB:c.-79A>G^/β^N^ | β^+^/β^N^ | 6 | 0.27% | 0.01% |
| β^HBB:c.130G>T^/β^N^ | β^0^/β^N^ | 4 | 0.18% | 0.01% |
| β^HBB:c.316-90A>G^/β^N^ | β^Unclear^/β^N^ | 3 | 0.14% | 0.01% |
| β^HBB:c.165_177delTATGGGCAACCCT^/β^N^ | β^0^/β^N^ | 3 | 0.14% | 0.01% |
| Taiwanese deletion/β^N^ | N/A | 2 | 0.09% | 0.00% |
| β^HBB:c.316-7C>A^/β^N^ | β^++ (silent)^/β^N^ | 1 | 0.05% | 0.00% |
| β^HBB:c.316-3C>T^/β^N^ | β^Unclear^/β^N^ | 1 | 0.05% | 0.00% |
| β^HBB:c.92+1G>T^/β^N^ | β^0^/β^N^ | 1 | 0.05% | 0.00% |
| β^HBB:c.-78A>C^/β^N^ | β^+^/β^N^ | 1 | 0.05% | 0.00% |
| β^HBB:c.79G>A^/β^HBB:c.79G>A^ | β^+^/β^+^ | 1 | 0.05% | 0.00% |
| β^HBB:c.126_129delCTTT^/β^HBB: c.-11_-8delAAAC^ | β^0^/β^+^ | 1 | 0.05% | 0.00% |
| β^HBB:c.316-146T>G^/β^N^ | β^+^/β^N^ | 1 | 0.05% | 0.00% |
| β^HBB:c.-78A>G^/β^HBB:c.-100G>A^ | β^+^/β^Unclear^ | 1 | 0.05% | 0.00% |
| Chinese (^A^γδβ)^0^/β^HBB:c.316-197C>T^ | ^G^γ(^A^γδβ)^0^/β^0 /+^ | 1 | 0.05% | 0.00% |
| β^HBB:c.316-197C>T^/β^N^ | β^++^/β^N^ | 1 | 0.05% | 0.00% |
| β^HBB:c.52A>T^/β^HBB:c.-100G>A^ | β^0^/β^Unclear^ | 1 | 0.05% | 0.00% |
| β^HBB:c.126_129delCTTT^/β^HBB:c.-100G>A^ | β^0^/β^Unclear^ | 1 | 0.05% | 0.00% |
| β^HBB:c.-137C>T^/β^N^ | β^++^/β^N^ | 1 | 0.05% | 0.00% |
| β^HBB:c.93-2A>G^/β^N^ | β^0^/β^N^ | 1 | 0.05% | 0.00% |
| β^HBB:c.380T>G^/β^N^ | β^+^/β^N^ | 1 | 0.05% | 0.00% |
| β^HBB:c.-31C>T^/β^N^ | β^Unclear^/β^N^ | 1 | 0.05% | 0.00% |
| β^HBB:c.-80T>C^/β^N^ | β^+^/β^N^ | 1 | 0.05% | 0.00% |
| Total |  | 2207 | 100.00% | 4.06% |

*Detection rate was calculated as: (Number of cases / Total NGS-screened population [N=54,357]) × 100%.

**Supplementary Table 5.** Genotype distribution and detection rates of β-thalassemia carriers identified by traditional cascade screening.

| **Genotypes** | **Phenotype** | **Cases** | **Constituent ratio (%)** | **Detection rate (%)*** |
| --- | --- | --- | --- | --- |
| β^HBB:c.316-197C>T^/β^N^ | β^0/+^/β^N^ | 150 | 37.69% | 0.40% |
| β^HBB:c.126_129delCTTT^/β^N^ | β^0^/β^N^ | 119 | 29.90% | 0.32% |
| β^HBB:c.-78A>G^/β^N^ | β^+^/β^N^ | 52 | 13.07% | 0.14% |
| β^HBB:c.52A>T^/β^N^ | β^0^/β^N^ | 41 | 10.30% | 0.11% |
| β^HBB:c.85dupC^/β^N^ | β^0^/β^N^ | 11 | 2.76% | 0.03% |
| β^HBB:c.217dupA^/β^N^ | β^0^/β^N^ | 6 | 1.51% | 0.02% |
| β^HBB:c.-79A>G^/β^N^ | β^+^/β^N^ | 5 | 1.26% | 0.01% |
| β^HBB:c.45dupG^/β^N^ | β^0^/β^N^ | 4 | 1.01% | 0.01% |
| β^HBB:c.79G>A^/β^N^ | β^+^/β^N^ | 3 | 0.75% | 0.01% |
| Chinese (^A^γδβ)^0^/β^N^ | ^G^γ(^A^γδβ)^0^/β^N^ | 2 | 0.50% | 0.01% |
| SEA-HPFH/β^N^ | N/A | 2 | 0.50% | 0.01% |
| β^HBB:c.126_129delCTTT^/β^HBB:c.-11_-8delAAAC^ | β^0^/β^+^ | 2 | 0.50% | 0.01% |
| β^HBB:c.-11_-8delAAAC^/β^N^ | β^+^/β^N^ | 1 | 0.25% | 0.00% |
| Total |  | 398 | 100.00% | 1.06% |

*Detection rate was calculated as: (Number of cases / Total traditionally-screened population [N=37,500]) × 100%.

**Supplementary Table 6.** Comparative analysis of α-thalassemia genotype distribution and detection bias between NGS-based universal screening and traditional cascade screening strategies.

| **α-thalassemia genotype** | **NGS-based Universal Screening (n=6258)** | | **Traditional Cascade Screening (n=840)** | |
| --- | --- | --- | --- | --- |
|  | **Cases** | **Constituent ratio (%)** | **Cases** | **Constituent ratio (%)** |
| --^SEA^/αα | 3349 | 53.52% | 620 | 73.81% |
| -α^3.7^/αα | 1757 | 28.08% | 130 | 15.48% |
| -α^4.2^/αα | 529 | 8.45% | 34 | 4.05% |
| α^HBA2:c.369C>G^α/αα | 250 | 3.99% | 11 | 1.31% |
| α^HBA2:c.427T>C^α/αα | 134 | 2.14% | 21 | 2.50% |
| α^HBA2:c.377T>C^α/αα | 55 | 0.88% | 3 | 0.36% |
| --^SEA^/-α^3.7^ | 41 | 0.66% | 13 | 1.55% |
| --^SEA^/-α^4.2^ | 14 | 0.22% | 5 | 0.60% |
| other | 129 | 2.06% | 3 | 0.36% |

Statistical analysis: Pearson's chi-square test showed a significant difference in genotype distribution between the two strategies (χ² = 157.46, df = 8, *P* < 0.001).

**Supplementary Table 7.** Comparative analysis of β-thalassemia genotype distribution and detection bias between NGS-based universal screening and traditional cascade screening strategies.

| **β-thalassemia genotype** | **NGS-based Universal Screening (n=2207)** | | **Traditional Cascade Screening (n=398)** | |
| --- | --- | --- | --- | --- |
|  | **Cases** | **Constituent ratio (%)** | **Cases** | **Constituent ratio (%)** |
| βHBB:c.316-197C>T/β^N^ | 831 | 37.65% | 150 | 37.69% |
| βHBB:c.126_129delCTTT/β^N^ | 629 | 28.50% | 119 | 29.90% |
| β^HBB:c.-78A>G^/β^N^ | 265 | 12.01% | 52 | 13.07% |
| β^HBB:c.52A>T^/β^N^ | 128 | 5.80% | 41 | 10.30% |
| βHBB: c.-11_-8delAAAC/β^N^ | 98 | 4.44% | 1 | 0.25% |
| βHBB:c.-100G>A/β^N^ | 56 | 2.54% | 0 | 0.00% |
| β^HBB:c.85dupC^/β^N^ | 36 | 1.63% | 11 | 2.76% |
| β^HBB:c.79G>A^/β^N^ | 30 | 1.36% | 3 | 0.75% |
| other | 134 | 6.07% | 21 | 5.28% |

Statistical analysis: Pearson's chi-square test showed a significant difference in genotype distribution between the two strategies (χ² = 40.47, df = 8, *P* < 0.001).

**Supplementary Table 8.** Detection Rates for Different Clinical Severity Categories.

| **Severity Category** | **NGS Couples (n=27,211)*** | **NGS Detection Rate (%)** | **Traditional Couples (n=18,750)**** | **Traditional Detection Rate (%)** | **Detection Rate Ratio (%)** |
| --- | --- | --- | --- | --- | --- |
| Double-negative | 19,374 | 71.20% | 234** | 1.25% | 1.80% |
| Single-positive | 7,160 | 26.31% | 669** | 3.57% | 13.60% |
| Double-positive | 677 | 2.49% | 259** | 1.38% | 55.40% |
| α-high severity | 122 | 0.45% | 60 | 0.32% | 71.40% |
| α-intermediate-high severity | 15 | 0.06% | 7 | 0.04% | 67.30% |
| α-intermediate severity | 163 | 0.60% | 47 | 0.25% | 41.90% |
| α-intermediate-low severity | 18 | 0.07% | 3 | 0.02% | 24.20% |
| α-low severity | 68 | 0.25% | 9 | 0.05% | 19.20% |
| β-high severity | 41 | 0.15% | 28 | 0.15% | 98.70% |
| β-low severity | 6 | 0.02% | 1 | 0.01% | 22.70% |
| Inter-type (α+β) | 246 | 0.90% | 104 | 0.56% | 61.40% |

*In the NGS cohort, 54,357 individuals underwent genetic testing, resulting in the genotype matching of 27,211 couples. **For the traditional cohort, couple matching outcomes (double-negative, single-positive, double-positive) are based on 1,162 couples who underwent genetic testing, while severity category detection rates are based on all 18,750 matched couples. Detection Rate Ratio = (Traditional Detection Rate / NGS Detection Rate) × 100%.
